# Supplementary material for: Anticoagulation-related complications and their outcomes in hemodialysis patients with acute kidney injury at selected hospitals in Ethiopia
Source: PLoS One. 2024 Dec 27;19(12):e0300301. doi: 10.1371/journal.pone.0300301 (PMC11676946; doi:10.1371/journal.pone.0300301)
Supplement: Attachment — Submitted filename: Response to reviwers.docx [file pone.0300301.s001.docx]

**MANUSCRIPT NUMBER: PONE-D-24-07202**

**MANUSCRIPT TITLE:** Anticoagulation-related complications and their outcomes in hemodialysis patients with acute kidney injury at selected hospitals in Ethiopia.

| RN | ACADEMIC EDITOR COMMENTS | AUTHOR RESPONSES |
| --- | --- | --- |
| 1. | Please ensure that your manuscript meets PLOS ONE's style requirements, including those for file naming. | Dear academic editor, Thank you for your guidance. We have reviewed and updated the manuscript to ensure full compliance with PLOS ONE's style requirements, including those for file naming. |
| 2. | We note that your Data Availability Statement is currently as follows: [All relevant data are within the manuscript and its Supporting Information files.] Please confirm at this time whether or not your submission contains all raw data required to replicate the results of your study. Authors must share the “minimal data set” for their submission. | Dear Academic Editor, thank you for the clarification on the Data Availability Statement. We confirm that our submission includes all data necessary to replicate the findings of our study, |
| 3. | Please upload a copy of Figure 1, to which you refer in your text on page 15. If the figure is no longer to be included as part of the submission please remove all reference to it within the text | Dear Academic Editor, thank you for bringing this to our attention. We have uploaded Figure 1 in the revised manuscript as separate file. . |
| 4. | Please review your reference list to ensure that it is complete and correct. If you have cited papers that have been retracted, please include the rationale for doing so in the manuscript text, or remove these references and replace them with relevant current references. Any changes to the reference list should be mentioned in the rebuttal letter that accompanies your revised manuscript. If you need to cite a retracted article, indicate the article’s retracted status in the References list and also include a citation and full reference for the retraction notice. | Dear Academic Editor, thank you for the reminder regarding the reference list. We have carefully reviewed all references to ensure they are complete, accurate, and up-to-date. |

***N.B:*** **RN**; Roll Number

| RN | REVIEWER 1 COMMENTS | AUTHOR RESPONSES | PN and LN |
| --- | --- | --- | --- |
| 5. | Is the manuscript technically sound, and do the data support the conclusions?  The manuscript must describe a technically sound piece of scientific research with data that supports the conclusions. Experiments must have been conducted rigorously, with appropriate controls, replication, and sample sizes. The conclusions must be drawn appropriately based on the data presented. | Dear reviewer, thank you for your feedback. Our study was conducted with rigorous scientific standards, including a multicenter observational design, consistent protocols across 1010 HD for 175 AKI patients, and real time data collection by trained personnel. We used multinomial logistic regression and descriptive statistics to identify key associations, such as between HD frequency, blood flow rates, and anticoagulation complications. Our conclusions are directly drawn from these findings, aligning statistical results with observed outcomes. We believe this demonstrates the technical soundness and data support for our conclusions. |  |
|  | Reviewer #1: Partly |  |  |
| 6. | Has the statistical analysis been performed appropriately and rigorously? | Thank you for your positive feedback regarding the statistical analysis. | - |
|  | Reviewer #1: Yes |  |  |
| 7. | Have the authors made all data underlying the findings in their manuscript fully available?  The PLOS Data policy requires authors to make all data underlying the findings described in their manuscript fully available without restriction, with rare exception (please refer to the Data Availability Statement in the manuscript PDF file). The data should be provided as part of the manuscript or its supporting information, or deposited to a public repository. For example, in addition to summary statistics, the data points behind means, medians and variance measures should be available. If there are restrictions on publicly sharing data—e.g. participant privacy or use of data from a third party—those must be specified. | We appreciate your acknowledgment of the data availability. | - |
|  | Reviewer #1: Yes |  |  |
| 8. | Is the manuscript presented in an intelligible fashion and written in standard English?  PLOS ONE does not copyedit accepted manuscripts, so the language in submitted articles must be clear, correct, and unambiguous. Any typographical or grammatical errors should be corrected at revision, so please note any specific errors here. | Thank you for your positive feedback regarding the clarity and language of our manuscript. | - |
|  | Reviewer #1: Yes |  |  |
| 9. | Please use the space provided to explain your answers to the questions above. You may also include additional comments for the author, including concerns about dual publication, research ethics, or publication ethics. (Please upload your review as an attachment if it exceeds 20,000 characters) | - Dear Reviewer 1, Thank you for your valuable comments. We have updated the Characteristics of HD Sessions section to include the number of patients who underwent heparin-free dialysis, along with the reasons for it, such as elevated bleeding risk or contraindications. - Citrate anticoagulation is not a common method of anticoagulation in the Ethiopian setting due to its limited availability, the need for regular and strict monitoring parameters, and our resource-constrained environment, despite its advantages for patients with AKI. - Thank you for your insightful feedback regarding the correlation between blood flow rate and clot formation in patients undergoing dialysis for acute kidney injury. We have clarified in our revised manuscript to emphasize both the direct and indirect factors contributing to reduced blood flow in this patient population. Thank you again for your valuable input. - Thank you for your comments. We have included details about the central venous catheters (CVCs) used in our study in the revised manuscript. Specifically, we utilized non-tunneled (temporary) hemodialysis catheters placed in the jugular or femoral vein sites. | P_20-21,_ L_305-307_  P_22,_ L_345-352_  P_14,_ L_205-207_ |
|  | Reviewer #1: I read with interest the paper by Kedir et al. about bleeding and clotting complications in HD patients treated for AKI. The paper has some limitations, mainly due to the challenging and different settings than usually observed in Western countries; I only suggest some revisions to improve the comprehension and generalizability of study results.  - The anticoagulation strategies greatly vary between centers; please indicate how many patients do not receive heparin, the reason, and the outcomes in this subgroup. At the same time, the authors can state the reason for the unavailability of citrate anticoagulation (difficulty in supplying? Costs? Both?) considering the advantages in AKI patients (see as example 10.3390/biomedicines11092570)  - The correlation between blood flow rate and clots may be direct (e.g., hemodynamic problems in the patients with low tolerance to the treatment) or indirect (e.g., problems in the vascular access limiting the flow rate favoring clot formation). Please specify the primary cause of reduced blood flow in patients with clotting and comment on it.  - If I understand correctly, all patients have central venous catheters as vascular access. Please include the characteristics of the CVC (materials, tunneled or not, temporary or permanent) and the site (jugular/femoral vein) because all these characteristics may influence the clotting risk. If possible, mention the machinery and technique adopted for the dialysis sessions (HD, HDF, hemofiltration). |  |  |

***N.B:*** **RN**; Roll Number, **PN**; Page Number, **LN**; Line Number

| RN | REVIEWER 2 COMMENTS | AUTHOR RESPONSES | PN and LN |
| --- | --- | --- | --- |
| 1. | Is the manuscript technically sound, and do the data support the conclusions?  The manuscript must describe a technically sound piece of scientific research with data that supports the conclusions. Experiments must have been conducted rigorously, with appropriate controls, replication, and sample sizes. The conclusions must be drawn appropriately based on the data presented. | Thank you for your positive feedback regarding the technical soundness of our manuscript. | - |
|  | Reviewer #2: Yes |  |  |
| 2. | Has the statistical analysis been performed appropriately and rigorously? | Thank you for your positive feedback regarding the statistical analysis. | - |
|  | Reviewer #2: Yes |  |  |
| 3. | Have the authors made all data underlying the findings in their manuscript fully available? | We appreciate your acknowledgment of the data availability. | - |
|  | Reviewer #2: Yes |  |  |
| 4. | Is the manuscript presented in an intelligible fashion and written in standard English?  PLOS ONE does not copyedit accepted manuscripts, so the language in submitted articles must be clear, correct, and unambiguous. Any typographical or grammatical errors should be corrected at revision, so please note any specific errors here. | Thank you for your positive feedback regarding the clarity and language of our manuscript. | - |
|  | Reviewer #2: Yes |  |  |
| 5. | Please use the space provided to explain your answers to the questions above. You may also include additional comments for the author, including concerns about dual publication, research ethics, or publication ethics. (Please upload your review as an attachment if it exceeds 20,000 characters) | Dear Reviewer 2, Thank you so much for your thoughtful review of our manuscript. We truly appreciate your positive feedback on the study’s relevance and the statistical analyses we employed. We acknowledge your comment about the language needing some polishing for better flow. We have carefully revised the manuscript to enhance clarity and readability throughout the text. | Revised MS |
|  | Reviewer #2: The manuscript by Kedir et al. “Anticoagulation-related complications and their outcomes in hemodialysis patients with acute kidney injury at selected hospitals in Ethiopia” looks interesting. This article aims to evaluate anticoagulation outcomes and related factors in hemodialysis (HD) patients with acute kidney injury (AKI) at selected hospitals in Ethiopia. Descriptive statistics were utilized to summarize the data from AKI patients undergoing HD during October 2021 to March 2022, and multinomial logistic regression analysis was employed to identify factors associated to clotting and bleeding. The authors found that clotting affected 19.4% of participants, while bleeding occurred in 15.4%, underscoring the importance of close monitoring. The manuscript is well written and provides enough strategies to fulfill its aims, though language may need some polishing for better flow. In conclusion, I support publication of this work and believe that it would be interesting for researchers working in the field. |  |  |
